# Reviewed article: Lopes LPN, Raimundo ACS, Caetano R, Lopes LC. Recalculation of the budgetary impact of risperidone use for Autism Spectrum Disorder in Brazil: a case study using measured demand data, 2017-2019. Epidemiol Serv Saude. 2025:34;e20240732.

**DOI:** 10.1590/S2237-96222025v34e20240732.d

**Published:** 2025-08-08

**Authors:** Cristiano Bertolossi Marta

**Affiliations:** Universidade do Estado do Rio de Janeiro, Fundamentos de Enfermagem

**Table:** 

Rating	Excellent	Good	Average	Below average	Poor
Interest	✓				
Quality	✓				
Originality	✓				
Overall	✓				

**Table:** 

Questionnaire	Yes	No	Not applicable
Does the manuscript contain new and significant information to justify publication?	✓		
Does the Abstract (Summary) clearly and accurately describe the content of the article?	✓		
Is the problem significant and concisely stated?	✓		
Are the methods described comprehensively?	✓		
Are the interpretations and conclusions justified by the results?	✓		
Is adequate reference made to other work in the field?	✓		

## Manuscript Structure

Length of article is: Adequate

Number of tables is: Adequate

Number of figures is: Adequate

Please state any conflict(s) of interest that you have in relation to the review of this paper (state “none” if this is not applicable): None

## Comments

Pesquisa relevante para sociedade e muito bem escrita dentro do contexto das avaliações Economicas em saúde.

Este estudo de caso mostrou importantes diferenças das estimativas das análises de impacto orçamentário presentes nos relatórios de recomendação, feitas durante a incorporação da tecnologia, exclusivamente através da demanda epidemiológica, em comparação com os gastos previstos a partir da demanda aferida no SUS.

Parabéns!! 

